# Syphilis and Chancroid, a Clinical Lecture

**Published:** 1885-04

**Authors:** W. D. Bizzell

**Affiliations:** Professor Practice of Medicine, Atlanta, Ga.


### SYPHILIS AND CHANCROID.

A CLINICAL LECTURE DELIVERED AT THE SOUTHERN MEDICAL COLLEGE.

BY W. D. BIZZELL, M. D.,
PROFESSOR PRACTICE OF MEDICINE, ATLANTA, GA.

Gentlemen: We present to you to-day two cases which illustrate quite prominently the characteristics of the two diseases under consideration.

First, we call your attention to B. H., age about forty. You will observe that he looks rather weak, anæmic, and somewhat haggard, as though suffering from physical pain, and he has that ashy, scurfy condition of the skin that in the colored race stands for pallor in the white race. On examining the genital organs you will observe three distinct sores, which are what are called phagadenic chancroids by French, English and American writers, and chancre by the German writers, who describe the initial lesion of the constitutional affection as syphilis. In the patient before you there appear three foul, ragged, ulcerated sores; one on the outside of the long prepuce, which looks shallower but unhealthy and sloughy; one transversely across the upper surface at the root of the penis, the ulcer involving the whole thickness of the skin with ragged edges and worm-eaten base; the third is in the inguinal fold, and has resulted from the absorption into one or more absorbent glands of the region, the septic matter of the sore causing suppurative destruction of the gland and the production of the deep, ragged and irregular shaped ulcer which you behold. Your reading, observation and the lectures you have already heard have taught you to look for two kinds of sores, the origin of which is distinctly venereal, one of which is purely local in character, exhausting all of its energies in a local destruction of tissue, the other constitutional, impressing itself on the blood and producing changes in that fluid and in the vital cell growth and tissue sensoral, interrupting the fine and delicate operations of building up the body with imperfectly elaborated material, or pouring into tissue or gland a mass of cells doomed to speedy destruction, whose disintegration may cause frightful ravages, and sooner or later bring death.

It is then of the utmost importance that the medical man be able to recognize and differentiate the two diseases. There is reason to believe that the local, ragged, ugly looking sores which you see in this case have been met with from the earliest periods of the world's history. You will observe that it is soft, has no induration at the base of the ulcer, is ragged, and has an appearance of being worm-eaten at the bottom.

Second, that it is multiple; also, you will observe that one of the sores has been produced by the suppuration of an inguinal gland. These are all important features in making the diagnosis of chancroid.

The next patient, C., I now present, and we will discuss the two together, and by confrontation and comparison we hope to impress upon you more clearly the more salient points in the appearance of the sores, the clinical history and pathological anatomy by which a differential diagnosis may be reached and clearly established.

We examine the genital organ. Retracting the prepuce you observe on its inner aspect, near the furrow at the base of the glands, a small surface somewhat raw, slightly raised above the surface, and when I grasp the part between my thumb and finger it produces the sensation that a flattened bullet or other foreign body might, so dense is the area embraced in the sore or initial lesion. As a sore it does not impress you as do the three ugly and shaggy sores of B. H. There is virtually no solution of continuity, no tender papillæ or nerve twigs exposed, and I can handle the part freely without provoking complaint on the part of the patient. The intelligence of these two persons is so low, and their sexual relations so promiscuous and careless that it is difficult to get a clear clinical history of these cases. B. H. tells us that he don't remember exactly how long since his first sore made its appearance on the prepuce, but that it was a little sore and gradually got deeper and wider, and pretty soon after he noticed a swelling in the groin, and about this time he also noticed a small sore commencing among the pubic hair just at the upper surface of the root of the penis; the swelling in the groin grew more and more pronounced uutil the skin became soft and undermined, burst, discharged its contents, and eventually become the ragged, raw surface which you now behold. This is about all he can tell us, except that he has had no treatment, not even to the extent of maintaining cleanliness.

The other man, C., seemed surprised when we expressed the desire to interrogate his genital organs; stoutly maintained there was nothing wrong there; that he had noticed a small sore there some time since, but that it was well now. What we found on the penis we have already described. On examination we find the inguinal glands on the side corresponding to the sore enlarged, indolent and apparently without a tendency to suppurate. What the patient complained of was a sore throat, but more especially of an atrocious pain located along the middle portion of the shaft of the right tibia.

The first man has a local sore or sores, which, though ugly and painful and sometimes slow to heal, will always be local, and unless they be inoculated with syphilis will never give him any trouble of a general or constitutional character. The treatment will also be purely local, except the administration of some general tonic to combat the anæmia and general debility which usually attend the more pronounced types of chancroid, particularly phagadena. At the close of the lecture we will etherize and then, having clipped off the pubic hair around, and thoroughly cleansed the parts, we will apply the fuming nitric acid to every part of the sores, keep them dusted with iodoform and lay over this pledgets of absorbent cotton softened with vasaline. This dressing renewed as often as is necessary, with strict cleanliness, and the prescription of some simple tonic, say tartrate of iron in infusion of gentian or columbo, will be all that is necessary, unless, perhaps a re-application of the nitric acid.

C., in the meantime, is patiently waiting for us to pronounce upon his case. As you have already no doubt decided, his trouble is constitutional; his sore is simply the initial point or avenue by which the contagious principle has found an entrance. On a careful examination we find scattered here and there little dry crusts of small size, and scaly dry condition of the skin. If he were white there would be a coppery hue developed round these points. Examining the throat, we see that particular lesion called mucous patches, raw, red places, deprived of the epithelial layer. We now examine the limb and find what is called a node along the crest of the tibia, which is exquisitely sensitive on pressure, and on getting warm in bed so tortures the patient that he instinctively throws off the cover and the sleep is so broken that he is wretched and depressed in consequence. This patient having been submitted to no treatment during the time since the contraction of the disorder, he illustrates very nicely the remnant of the hard sore initial lesion, the secondary symptoms on skin and mucous membrane, which show evidence of having passed the acme of eruption, and now he is developing on shafts of the tibia a node, symptom of the tertiary stage, or what is sometimes called the stage of sequelæ. Of course, to accomplish any good in this case the treatment must be constitutional. Though some writers still confuse the chancroid with the initial lesion of syphilis, it seems to me that, after a careful study of two such cases as those, such a mistake would be impossible. The syphilis, as in this case, commences as a small, insignificant, indurated point, too trifling to merit much attention from the patient. What is the nature of the new material which constitutes the induration of the initial lesion? A proliferation of cell growth, exactly resembling those of embryonal growth, with this as a nidus, slowly along the absorbents the poison travels, until the first chain of glands in the lymphatics is reached, when an indolent engorgement takes place, but it does not stop here to destroy the gland with a destructive inflammatory disintegration, as would be the case in chancroid, and has actually happened in the case of chancroid before you, but traveling on into the larger lymphatic ducts the baleful poison is finally poured into the blood current. Then it is that we have those eruptions on skin and mucous surfaces, which are denominated the secondary lesions, constitutional manifestations, usually attended with more or less febrile reaction, still maintaining its peculiar characteristic of plastic inflammation, attended by evolution of embryonal cells.

The local sore of chancroid, if strongly irritated by caustic applications, may, from the effusion of lymph, develop an indurated base, but usually you will learn from the patient that he has been burning it with a cigar stump, cigar ashes, copperas, or a stick of lunar caustic. By simple soothing treatment for a few days, the induration will disappear, unless syphilis has been inoculated into the base of the chancroid, an accident which you should not lose sight of.

On the other hand, the initial lesion of syphilis, if unduly irritated by caustics, etc., will assume an angry and threatening look, and the area of induration will be vastly increased. An interesting case of this kind I saw a year or two since, with my friend Dr. Divine, of this city. Under the advice of a *physician* in the employ of a well advertised blood remedy, he had been making *daily applications* of the solid nitrate of silver, of course, taking the panacea at the same time. The glans penis was almost entirely destroyed, and the extremity of the penis was hard and bulbous like an onion root, in fact, resembled much in appearance, certain varieties of cancer of this organ. The doctor and I had no difficulty in recognizing the true nature of the trouble, as the secondary, copper-colored blotches covered the entire body. Soothing treatment of the sore, and constitutional treatment of the man, enabled him to go to work in two weeks and rescue his family from starvation, after three months of enforced idleness. One of the curious points in connection with chancroid is, that the poison, though virulent and destructive at the point of inoculation, and also in the first lymphatic gland it reaches along the absorbents, ususually ending in a destruction of the gland, but, strangely enough, its power for evil there stops, and no general infection follows, though the poison may be inoculated in several places and give rise to mullipe sores, as you see in the case of B. H.

After the chancroid and syphilis had been differentiated, mainly by the labors of the French school, and the confusion of two centuries cleared up, the next question to determine was, whether the chancroid was a sore, possessing a peculiar and special infecting poison, or was it simply pus inoculated under the skin ? Thousands of intelligent physicians still maintain that it is a poison *sui generis*, but the general tendency of the medical profession at this time is to regard them as produced by the inoculation of pus, scarcely, if at all, modified from pus under other circumstances. I do not think that the last is altogether tenable, although I must say that the change in the nature of the pus is a subtile one, and in my opinion, more likely chemical than from the development in it of a zymotic poison or living germ. For we do know that the use of strong chemicals or escharotics will stop the local destruction of chancroid, and the sore will lose the peculiar and characteristic appearance which we here described, and under soothing applications and cleanliness fill with granulations and heal. The gland envolvement in chancroid, which suppurates in nearly all cases, should be punctured early by a small opening, and the contents broken up with a grooved director and squeezed out, and the pus cavity thoroughly cauterized by wrapping cotton on a sliver of wood and saturating with nitric acid. If so handled they do not result in those ugly, ragged sores, which are so slow to heal in this locality.

As to the proper treatment of the man with syphilis, thousands of books have been written in the last 400 years on the subject, and medical opinion has been swayed like a pendulum, first this way and then that way; first mercury, and then no mercury; but within the last few years we have reached a common sense basis, and also know more of the true pathology of the disease, and comprehend far more clearly the action of drugs and what they can accomplish. As we have said, the pathology of syphilis is an abnormal growth of embryonal cells—first in the skin and mucous surfaces, and subsequently in the deeper structures of periosteum and bone, and subsequently any tissue of the body is liable to be invaded.

Any treatment to be successful in syphilis must be what is denominated alterative, and also tonic; alterative, to break up and remove the adventitious new cell material, and tonic in the sense of restoring and creating anew the red blood corpuscles, and getting rid of the vicious tendency to cell proliferation.

The experiments of Dr. Keye, of New York, show that when the patient takes small doses of mercury in an easily assimilable form, if samples of his blood be examined microscopically and the red discs counted in a germ, given area the micrometer there is a steady increase in their number; this corresponds exactly to the clinical experience of the intelligent practitioner. So that now it is universally conceded that to attack and destroy the very root of the evil in syphilis, which lies back of its morbid anatomy and originates it, just as a vicious and evil will precedes and determines a crime, there is nothing that can be compared to some of the preparations of mercury. But when the disease has reached the tertiary stage, and large masses of cells are being poured out in contact with bone, in the brain, or other structure of the body, producing that gross anatomical lesion which you see in this case over the shaft of the tibia, called a gummatta, some more active eliminator is demanded. Then iodide of potassium will do more than anything else that you can use to cause the disappearance of the morbid deposit. The gummy tumor developed in the brain or on the inside of the calrarium may produce the most atrocious headache, followed by convulsions, epileptiform insanity, paralysis, coma and death, then it is that the heroic use of potassium will serve you well and come to your patient like a blessed evangel in his dire extremity. In such cases the drug must be pushed to the last extremity of toleration. A patient growing constantly worse under small doses will show almost immediate improvement when you give him one drachm three times a day; and you can truly say that you have averted death. In the case before you we will give what is known as the compound treatment. We will give him about thirty grains of the iodide of potassium combined with about the fortieth of a grain of the bichloride of mercury, three times a day, and this we will keep up for a long time, until all of the manifestations of syphilis have subsided. After this he should have a prolonged course of mercury in very minute doses, not enough at any time to produce salivation, but enough to get the tonic and alterative effect.

The reason why so many cases of syphilis recur after apparent cure, is that treatment is abandoned too early; the temporary morbid anatomy is removed, but the evil and vicious tendency to such cell growth is not corrected. To be successful we should continue the treatment of C. for at least eighteen months on this plan. Though I have no doubt as soon as all symptoms disappear and he ceases to feel any inconvenience from the disease, he will cease to attend the clinic, and in all probability we will be called upon to treat him for a relapse of the trouble. But such is life, and such is the medical experience with the average patient.

